# ‘Localism and intimacy, and… other rather imponderable reasons of that sort’: A qualitative study of patient experience of community hospitals in England

**DOI:** 10.1111/hsc.14083

**Published:** 2022-11-03

**Authors:** Deborah Davidson, Iestyn Williams, Jon Glasby, Angela Ellis Paine

**Affiliations:** ^1^ Health Services Management Centre School of Social Policy University of Birmingham Birmingham United Kingdom; ^2^ School of Social Policy University of Birmingham Birmingham United Kingdom

**Keywords:** community hospitals, England, patient experience, research

## Abstract

Debates over the value and contribution of community hospitals are hampered by a lack of empirical assessment of the experience of patients using these services. This paper presents findings from a study which included a focus on patient and family‐carer experiences of community hospitals in England. We adopted a qualitative design involving nine case study hospitals. Data collection included interviews with patients (*n* = 60), carers (*n* = 28) and staff (*n* = 89). Through patients and carers highlighting the value of community hospitals feeling ‘close to home’, providing holistic and personalised care and supporting them through difficult transitions, the study confirms the importance of *functional* and *interpersonal* aspects of care, while also highlighting the importance of *social* and *psychological* aspects. These included having family, friends and the community close, maintaining social connections during periods of hospital treatment, and feeling less anonymous and anxious when attending the hospital due to the high levels of familiarity and connectedness. Although the experiences uncovered in this study were not uniformly positive, patients and carers placed a high overall value on the care provided by community hospitals, often arguing that these were distinctive when compared to their experiences of using other health and care services. The study suggests the need to weigh the full range of these dimensions of patient experience—functional, interpersonal, social and psychological—when assessing the role and contribution of community hospitals.

**What is known about this topic?**

- Community hospitals are an historic, and diverse, feature of the healthcare landscape.
- There is little existing evidence focusing specifically on patient and carer experience of community hospitals.
- Wider patient experience research point to the importance of function and inter‐personal aspects of care.

**What does this paper add?**

- This study provides original empirical evidence on the importance of community hospitals being ‘closer to home’, providing personalised and holistic care, and supporting users through difficult transitions.
- We offer a new conceptualisation of patient and carer experience, which focuses on the interaction of function, interpersonal, social and psychological aspects.
- We conclude that community hospitals provide an embedded, relational model of care, which results in distinct patient and carer experiences.

## 1 INTRODUCTION

Community hospitals are a longstanding feature of the health and care landscape, both in the United Kingdom and internationally, and have remained remarkably durable in the face of multiple system re‐organisations (Pitchforth et al., 2017). However, this resilience masks some vulnerability. System integration, centralisation and emphasis on economies of scale are frequently accompanied by the proposed closure or downgrading of community hospital services. As Jones and Exworthy (2015) argue, the claims made for the efficiency and effectiveness of such changes often rest on inconclusive and partial evidence, which is regularly challenged by those campaigning to retain local hospital services (Williams et al., 2021). These evidence deficits include a lack of empirical assessment of the experience of patients using community hospital services. While there is a growing body of evidence on patient experience of various health and care settings, relatively little focuses specifically on community hospitals.

This paper explores patient and family‐carer experiences of community hospitals in England. The data were collected as part of a wider study into the profile, characteristics, patient experience and community value of community hospitals in England (Davidson et al., 2019), which included in‐depth interviews with patients and family‐carers in nine case studies. From our analysis, we propose a new conceptual framework for understanding patient and carer experiences of community hospital care. The framework links patient and carer accounts of community hospitals (e.g. as being ‘close to home’, providing personalised and holistic care, and supporting them through difficult transitions) to a differentiated and integrated focus on functional, interpersonal, social and psychological dimensions of care. Together, we suggest this points to an embedded, relational model of care within community hospitals.

### 1.1 Research setting: The community hospital sector in England

In England, community hospitals have been part of the healthcare landscape for more than 160 years, first emerging as small, predominantly rural ‘cottage’ hospitals. These were typically under the auspices of general practices (GPs) and provided inpatient beds and operating facilitates (Loudon, 1972), before evolving to also offer preventative and curative services (Dawson, 1920). Following the establishment of the National Health Service (NHS) in 1948, the government declared its preference for larger hospitals providing higher quality care, as encapsulated in the somewhat disparaging words of then UK Minister of Health Aneurin Bevan, quoted in the title of this paper (Bevan, 1946). However, the cottage, or ‘community’ hospital sector remained largely in place through the ensuing decades, albeit ‘re‐purposed’ in various ways, including: as a ‘bridge’ between primary and secondary care; as an alternative to larger hospitals for supporting people with complex needs (Hadridge, 1997); as a place for providing integrated health and social care (Department of Health and Social Care, 2003); as a ‘hub’ for the provision of outpatient clinics and admission prevention (Department of Health and Social Care, 2006), and; as part of an overall strategy for providing care closer to home (Department of Health and Social Care, 2008). While the services provided by community hospitals varied during this period, core elements included inpatient beds, outpatient clinics and minor injury units and facilities in which GPs and interdisciplinary teams worked together to support patients and their families, and to rehabilitate patients to return home (Willavoys & Crowther, 2013). Similar trends have been observed elsewhere. For example, a review of community hospitals in high‐income countries describes their role at ‘the boundary of primary care, acute hospital care and nursing home care’ and as providing ‘the full spectrum of service provision from preventative and primary care, to inpatient surgical or medical care’ (Winpenny et al., 2016).

In recent times, the place of community hospitals in England has become more unstable, as a combination of regional re‐organisations and workforce shortages have fragmented provision through two divergent trends: the closure of community hospitals and reduction of inpatient beds in some parts of the country; contrasting with the development of new community hospitals or hubs in others (Davidson et al., 2019, anonymised).

### 1.2 What is known about patient experience of community hospitals?

These contrasting developments reflect local system factors, such as arrangements for planning and funding, as well the presence of powerful individual and organisational actors within the healthcare landscape. They are also, arguably, made possible by the absence of robust evidence on the role and value of community hospitals. This is compounded by the high levels of heterogeneity among community hospitals. Indeed, there is no agreed definition of what constitutes a community hospital, especially when considered across international health contexts (Pitchforth et al., 2017). Despite claims and counter claims about the role and contribution of community hospitals, there is a notable lack of systematic research to inform policy (Davidson et al., 2019). There is a particular dearth of evidence on patient and carer experience. While a small number of high‐quality studies do exist, these tend to focus on inpatient services only and to rely on satisfaction surveys, rather than more in‐depth explorations of patient experience.

What the literature—from England and beyond—does suggest is the importance of three broad elements when assessing patient experience of community hospitals.

#### 1.2.1 Environment and facilities

Several studies of community hospitals identify important features of the environment and facilities which affect patient experience. These can be thought of as *functional* aspects of care. For example, studies find that patients value characteristics such as: close proximity to family and friends; the opportunity to interact with patients from the same geographical location; a homely and friendly atmosphere; an orientation to older people; high levels of cleanliness; availability of single room accommodation, and; the quality, choice and presentation of food (Clegg, 2003; Green et al., 2008; Lappegard & Hjortdahl, 2014; Payne et al., 2007; Small et al., 2007). By contrast, some patients report that community hospitals can be noisy environments (Lappegard & Hjortdahl, 2014; Small et al., 2009), and some inpatients report long periods of boredom (Payne et al., 2007; Small et al., 2009; Trappes‐Lomax & Hawton, 2012).

#### 1.2.2 Care delivery

Studies also report on the way that care is delivered within community hospitals. For example, inpatients often compare community hospital care favourably to acute care with regard to information sharing, continuity and the potential for longer lengths of stay (Department of Health, 2006; Green et al., 2005, 2008; Small et al., 2007, 2009). However, rehabilitation and ongoing needs are reported as not always being met on discharge (Trappes‐Lomax & Hawton, 2012). These can be considered as *functional* and *interpersonal* aspects of care.

#### 1.2.3 Staff

A smaller number of studies highlight the importance of staff, and relationships between staff and patients, to patient experience. Community hospital staff are often perceived more positively than those at larger hospitals, and associated with more personalised care (Green et al., 2005, Green et al., 2008; Payne et al., 2007;Small et al., 2007, 2009). However, at times patients report a lack of confidence in the technical skills of some staff and a preference for acute hospitals when requiring more complex medical care (Lappegard & Hjortdahl, 2014; Payne et al., 2007). Again, this points to both *functional* and *interpersonal* aspects of care.

Notwithstanding these studies, the evidence base remains underdeveloped, focusing primarily on the functional aspects of care, and using limited data collection approaches. Evidence of patient experience in other health and care settings (e.g. acute hospitals), however, provides useful insights. Bridges et al. (2010) argue that patients' and relatives' narratives rarely focus on the functional or technical aspects of the services they receive, instead foregrounding relational and interpersonal aspects of patient experience. Similarly, previous research into older people's experience of moving across service boundaries (Ellins et al., 2012), found that health and social care services often focused on the physical aspects of transition, whereas older people themselves tended to talk about transition in terms of the *psychological* changes in their identity or sense of self, and *social* changes in their relationships with partners, family and friends.

Overall, these limitations in the empirical literature on patient experience of community hospitals risk leading to reductive accounts of their contribution. There is a need to develop empirically and theoretical informed models of patient experience of community hospitals to properly assess their role and contribution in a constantly changing health and social care landscape.

## 2 METHODS

We draw on findings from a large multi‐methods study of the role, purpose and contribution of community hospitals in England (Davidson et al., 2019). The study was granted ethical approval by the Wales Research Ethics Committee 6 (reference number: 16/WA/0021). As one part of this wider study, we sought to explore and understand patient and family‐carer experiences of community hospitals, and to identify the factors that influenced them.

We focus on data from nine qualitative case studies of community hospitals, undertaken between November 2015 and February 2017. The case studies were selected from a dataset of 296 community hospitals in England (see Davidson et al., 2019) to reflect the diversity of community hospitals in terms of location across England, number of beds, service provision, models of ownership, levels of voluntary income and population deprivation (see Table 1).

**TABLE 1:** Profile of case study community hospitals

Case study	Owner (provider)	Available beds	Urban/ rural code^a^	MSOA IMD^b^
CH1: A classic community hospital providing ‘cradling to grave’ services, through inpatient beds, MIU, maternity unit, renal unit, X‐ray and outpatient services	NHS (NHS)	19	2	27.48
CH2: A large community hospital, with inpatient beds, day surgery, maternity, diagnostics and an extensive outpatient services	NHS (NHS)	37	1	28.7
CH3: A classic community hospital providing ‘cradling to grave’ services, through inpatient beds, MIU, maternity unit and outpatient services	NHS (CIC)	33	3	4.68
CH4: A mixed community hospital, with inpatient beds, a mental health facility and various outpatient clinics	NHS (CIC)	31	3	3.01
CH5: A classic community hospital providing inpatient beds, MIU, X‐ray, day care centre and outpatient services	NHS (NHS)	19	2	14.78
CH6: A small community hospital, limited to inpatient beds and one outpatient service	NHS (NHS)	22	3	12.84
CH7: A relatively small community hospital, with inpatient beds and a range of community and outpatient services, situated within a wider health and care campus	Charity (NHS)	13	2	33.04
CH8: A small community hospital, within inpatient beds, a limited range of community and outpatient services and space for the GP surgery.	NHS (NHS)	9	1	21.21.
CH9: A relatively large community hospital, providing ‘cradle to grave’ services including inpatient beds, MIU, X‐ray, clinical decisions unit, day hospice and extensive range of community and outpatient services.	NHS (NHS)	28	1	19.04

^a^ Based on the Office of National Statistics rural/urban classifications, where 1 is the most rural and 6 is the most urban.

^b^ Middle Super Output Areas in the Index of Multiple Deprivation (IMD): official measure of relative deprivation **for** small areas (or neighbourhoods) in England.

### 2.1 Sampling and recruitment

Each of the case studies involved interviews with various stakeholders including staff, volunteers, patients, carers and community members. In this paper, we draw directly on the interviews with patients, family carers and staff, although our analysis of this sub‐set of data is informed by and triangulated with our analysis of the wider dataset.

Patients and carers were purposively sampled, with efforts to ensure a mix of demographics (particularly gender), care pathways (particularly ‘stepped‐up’ via GP referral and ‘stepped‐down’ via acute hospital discharge) and services used. We sampled current inpatients, those who had been discharged recently, and long‐standing outpatients from a range of clinics. Potential participants were identified and written to by hospital staff, with a request to participate, an information sheet and an opt‐in consent form. Replies were sent directly to the study team, with full consent provided prior to commencement of the interview. Our final sample across all sites included 60 patients and 28 carers.

### 2.2 Data collection

Lessons from previous studies show that gathering evidence on patient experiences in the form of stories can enhance their depth and richness (Ellins et al., 2012). Each of our interviews began with a very open question inviting patients to describe their experience of the community hospital. This question was accompanied by a visual representation of factors found in previous research to have shaped patient experience, to prompt people's thoughts, memories and connections. Once participants had reached the end of their accounts, researchers used a series of prompts to follow‐up on aspects not already covered. To support analysis, at the end of the interviews, we asked respondents to complete a short pro forma to gather basic demographic and service information. Interviews with patients lasted, on average, 70 min.

In‐depth interviews were also conducted with 28 family carers in order to explore their experiences of community hospitals. In most cases, we interviewed carers of patients who had also been interviewed. In some cases, carers were not directly linked to patients involved in the study (e.g. some were reflecting on the experience of caring for a patient who had recently died). The focus of these interviews was on the experience of being a carer of someone at the hospital, with topic guides mirroring the pattern of the patient interviews by asking respondents to tell us their story of using the hospital, followed up with a series of prompts and additional wider questions. Interviews with family carers lasted, on average, 60 min.

Using in‐depth interviews enabled us to draw out not only what happened, but also how those experiences made patients and family carers *feel* about community hospital care (Cleary et al., 1992; Coulter & Cleary, 2001). Within our analysis and reporting, we aim to reflect an experience‐centred voice, capturing ‘meaning’ in the stories told.

We also interviewed community hospital staff as part of the wider study (*n* = 89). Although covering a wider range of topics, these interviews included questions about perceptions of patient experience, and therefore were of relevance to this paper, albeit we treated them as secondary to the testimony of patients and family carers in the development of our analysis. Interviews with staff lasted, on average, 60 min.

### 2.3 Data analysis

All interviews were digitally recorded (except for two, due to respondent preference), transcribed verbatim and assigned a unique identifier before being imported into NVivo11 software. We conducted thematic analysis, guided by Clarke et al. (2015) six step process which involved three members of the research team reading, re‐reading and inductively coding a sample of transcripts, and bringing this into conversation with concepts from the literature, to produce a jointly constructed draft coding frame. This was then tested, refined, reordered and grouped into themes, before the final version was applied across the whole dataset. Processes were put in place to ensure consistency across the team, including checking each other's coding practices. Themes were further validated during subsequent stages of analysis and reporting, and early findings were discussed with the wider research team and with national and local stakeholders.

Within the reporting of our findings, all sites and individuals are anonymised to ensure participant confidentiality. Unique identifiers are provided for each respondent quoted, denoting the community hospital (CH 1, 2, 3, etc.) and whether they are patients (P), carers (CA) or staff members (S). In the rest of the paper, the perspectives of these three groups are presented concurrently under each substantive theme, rather than as discrete standpoints presented in turn. This reflects the complexity of the sample with, for example, many current patients having previously been carers or hospital staff, and many staff having experienced the community hospital as patients or family carers. It also reflects the congruence of views across stakeholder groups regarding key features of patient and carer experiences.

We initially aimed to triangulate qualitative findings with existing hospital‐level data on patient experience (e.g. the NHS ‘Friends and Family Test’). However, these were not consistently accessible, and so while we collected and considered such data when it was available, we do not report on it here.

### 2.4 Patient and public involvement

Patient and public involvement was integral to all stages of the study. At the national level, 13 board members of the Community Hospitals Association (CHA) co‐produced the initial research proposal, with two members continuing on the study steering group. At a local level, reference groups were established to bring local stakeholders together to steer, support and inform the case study research.

## 3 FINDINGS

Table 2 profiles the interview sample, indicating a skew towards older age groups and a higher proportion of females in each category.

**TABLE 2:** Interviewee sample details

Characteristics	Number (%)
Patients (*n* = 60)	
Age	
18–39	1 (2%)
40–59	0 (0%)
60–79	25 (42%)
≥80	29 (48%)
Unspecified	5 (8%)
Gender	
Female	38 (63%)
Male	22 (37%)
Other	0 (0%)
Hospital	
1	6 (10%)
2	8 (13%)
3	5 (8%)
4	9 (15%)
5	7 (12%)
6	7 (12%)
7	7 (12%)
8	6 (10%)
9	5 (8%)
Carers (*n* = 28)	
Age	
18–39	0
40–59	2 (7%)
60–79	10 (36%)
≥80	6 (21%)
Unspecified	10 (36%)
Gender	
Female	19 (68%)
Male	9 (32%)
Other	
Hospital	
1	3 (11%)
2	2 (7%)
3	3 (11%)
4	2 (7%)
5	5 (18%)
6	2 (7%)
7	3 (11%)
8	3 (11%)
9	5 (18%)
Staff (*n* = 89)	
Gender	
Female	77 (87%)
Male	12 (13%)
Other	0 (0%)
Hospital	
1	9 (10%)
2	13 (15%)
3	3 (3%)
4	9 (10%)
5	13 (15%)
6	5 (6%)
7	10 (11%)
8	15 (17%)
9	12 (13%)

In our study, patients and family carers were generally positive when describing their experiences of community hospitals. Three clear themes were emphasised within respondents' accounts as being key to these experiences: being ‘close to home’; providing ‘personalised and holistic’ care and ‘supporting difficult transitions’. In the discussion section, we link these themes to our conceptual framework.

### 3.1 Close to home

Many patients and carers talked about community hospitals as not really being ‘*like a hospital*’; they said *‘it's closer to home’* in a number of ways. First, the local nature of community hospitals meant that they were often literally close to home, particularly when compared to acute services. Patients and carers valued having a local hospital which they felt was convenient, typically with only short distances to travel and accessible parking. Convenience was important for outpatient appointments, particularly for those who had to attend clinics regularly for relatively small procedures, as they enabled people to maintain their independence and self‐reliance:

‘That was that much more convenient and in fact I can drive myself here … To do it here makes a big difference in the life of somebody of my age [89 years] and ability to get around and that sort of thing.’ (CH1, P01)

Being conveniently located (alongside often flexible visiting hours) also enabled family members to visit regularly, which was particularly valued.

However, some community hospitals were located just outside of a village or town, making them feel somewhat remote and isolated. Moreover, some inpatients who had been referred to a community hospital from outside the local area talked about feeling a long way from home even when care was experienced as good. As an increasing proportion of inpatients within community hospitals are stepped‐down from acute services (rather than stepped‐up from primary care) and come from outside of the local area, this may become an increasing concern (Davidson et al., 2019).

Being physically close to home, however, meant more than convenience. Many of the community hospitals were deeply embedded in their local communities; staff were often drawn from the local community, and local communities had often been involved in supporting the hospital over generations, engendering a deep sense of connectedness for many patients and family carers, and a strong sense of familiarity. This ‘known‐ness’ was fundamental to many people's experiences of community hospital, and often held as a point of contrast to acute services, with important implications for well‐being. Family carers, for example, described the immediate and pronounced impact on their family member's mood once they were informed of discharge into the community hospital. Being able to attend a small, local hospital where they were known appeared to relieve patient stress: *‘you don't get tensed up about coming here.’* It also eased fears and anxieties for family carers:

‘I just knew that he was safe and for me that was huge. I was trying to work, I was trying to sort out what I was going to do with him, I was trying to sort out my father's palliative care … They just seemed to understand here that that's what we needed.’ (CH3, CA01)

However, while for most people ‘being known’ was a positive factor, for some patients there was concern that ‘*everyone knows everyone else's business’*, with a risk of compromising patient confidentiality.

The natural and built environment and atmosphere of community hospitals was also talked about by patients and carers as being *‘homely’*. For example, community hospitals were often (relatively) small, light and airy, and based within generous ground:

‘It is unique […] you go out into the day room and you look across those fields, you know, it's bright and airy and there's no sort of closed corners or anything. It doesn't feel like a hospital does it?’ (CH6, S05)

Comparisons were made with larger (acute) institutions, with the home‐like environment of community hospitals reported as making them feel more familiar, less intimidating, less stressful and more reassuring. This was particularly valued by patients who were older, frail and confused, especially when they were dying.

However, not all patients found all aspects of the environment positive. Issues such as night‐time noise from doors or a sluice disturbed some people's sleep. Some also described feeling isolated in single rooms and missing the social interaction of larger wards. In addition, some younger people associated community hospitals with an ‘old people's home’ and felt alienated because of their age.

### 3.2 Personalised and holistic

Community hospitals were seen as providing individualised, holistic, rehabilitative care. This was facilitated through a range of co‐located services, the fostering of multi‐disciplinary team working, the (frequent) involvement of patients' own GPs within the hospitals and/or staff that were known to patients, and an ethos which encouraged the time and space for staff (and volunteers) to work with people as individuals. This personalised approach extended to domestic and catering staff, and in community hospitals with their own kitchens patients particularly valued the food being cooked on site and served to them:

‘I had the same thing every morning so (laughing) it wasn't as if they needed to ask every morning, now would you like your white toast with no butter and just marmalade? Yes, please. Black tea? Yes, please. And they would remember.’ (CH1, P03)

For family carers, the dignity and respect given to their older relatives were important:

‘The respect and dignity they gave to my grandma was a huge thing for my mum and my aunty […]. I feel the respect that's shown to patients on the ward is – you can't compare it to anywhere.’ (CH9, S04)

The scale of community hospitals and the relatively small number of in‐patients were seen to further enable a personalised experience:

‘The whole thing was so much nicer and easier rather than if she'd have gone into the [acute hospital]. She'd have just been one more elderly person in a great big ward.’ (CH1, CA06)

As the above quote illustrates, direct comparisons were made with acute settings. There were numerous examples cited of community hospital staff identifying and addressing issues that had not been picked up during patients' earlier admissions to acute hospitals.

Patients also gave accounts of apparently tailored and personalised approaches to rehabilitation. A woman who spent 3 weeks in her local community hospital recovering from surgery having badly broken her pelvis, described how staff steadily supported her rehabilitation:

‘From the moment I got here they made me feel as though they were here to make me better and they were here to help me progress forward; it's not, ‘Oh, you're here. You can just sit and do nothing.’ […] If you're capable you will go and have your own shower and wash your hair and do all those sorts of things. And each day they praise you for achieving something new […] every time you achieve something you get the feeling that they're pleased for you, and I think that's vital and that again is the building of the confidence for people to go home.’ (CH7, P05)

However, time, and what to do with it, was a significant feature of daily inpatient life, and some patients, and carers, commented on a lack of social stimulation: *‘*[it's] *pretty boring laying here all day.’* The lack of ‘things to do’ was observed across a number of case study sites, albeit some good examples of social stimulation were identified. This raises a question as to whether more could be done to support social interaction between patients, albeit these complaints are unlikely to be unique to community hospitals.

### 3.3 Supporting transitions

Given community hospitals' focus on rehabilitation, a significant amount of time and effort was invested in supporting people to return home, following an inpatient stay. One patient talked about her home visit, and how staff assessed, supported and encouraged her, to understand how well she could cope at home:

‘Well, the home visit was about three or four days […] before I was discharged […] I did walk up the steps…they measured the height of the loo and looked at the shower and the kitchen and had me walking with the Zimmer. So they then had me going round the kitchen, ‘furniture walking’, they called it. (Laughing) […] you'd got someone there to see how you coped and offer you advice.’ (CH1, P03)

Interviewees reported that staff recognised the importance of involving family members and worked to build a good relationship with them, arranging meetings at the beginning and over the duration of the stay, to inform and involve them in what was likely to happen.

However, discharge could be a source of tension between staff, patients and family carers. Sometimes this was due to patients not wishing to return home, or because discharge had to be delayed due to patients not having sufficient family support and/or delays in arranging social care. At other times, pressure from acute hospitals to take people who no longer needed acute care (stepped‐down) meant that staff had to juggle priorities and this pressure then could be transferred to family carers who would find themselves responsible for caring for their relatives before they felt able to cope:

‘The pressure came because the [acute hospital] was on black so they were under pressure for beds here […] but I knew that if I didn't stand my ground that we were totally out on a limb. He was in hospital, I knew that he was being cared for, but as soon as I allowed him to be discharged, then I was on my own, so that was really difficult. I sat in meetings with five or six people just saying ‘No’.’ (CH3, CA01)

For many older people, the accident or illness that led to their admission to a community hospital often triggered a major life event, which was emotionally traumatic and a major psychological undertaking, and some had to come to terms with the likelihood that they would not return to their family home. There were many examples of staff working with patients to build their confidence, to support them through difficult transitions. However, given the substantial number of patients who were experiencing life transitions and who appeared shaken by those events or an unknown future, there was little explicit evidence of mental health needs being integral to inpatient care practice: we observed little formal assessment of, and work with, anxiety and depression. Efforts appeared to focus on distracting people from their general anxieties and concerns, rather than directly addressing people's psychological, emotional and mental health.

## 4 DISCUSSION

Cutting across these different accounts of patient experience are four dimensions that are key to understanding patient and carer experiences: functional, interpersonal, social and psychological (see Table 3). As noted, previous studies have tended to focus on three broad elements when assessing patient experience of community hospitals: environment and facilities, care delivery and staff. This work typically concentrates on the functional and interpersonal aspects of patient experience (Doyle et al., 2013; Glenn & Cornwell, 2011), and their importance is confirmed in our study. For example, functional, particularly environmental, features of community hospitals were fundamental to patient and family carer experiences. However, while these features were part of what made community hospitals feel ‘closer to home’, being closer to home went beyond convenience to represent an environment that was often familiar, known, reassuring and nurturing, particularly for local patients and their families.

**TABLE 3:** Illustrations within a framework of patient experience in community hospitals

	Functional	Interpersonal	Social	Psychological
Close to home	Convenience—short distance to travel. Homely buildings and gardens	Local staff and volunteers often known to patients and carers	Regular visits by family, friends and wider community members. Shared meal times	Familiarity—eased anxiety
Personalised & holistic	Co‐located services and teams	Own GP. Time for staff and volunteers to get to know you.	Activities provided by (local) volunteers	Feeling known and cared for
Supporting transition	Proximity facilitates home visits	Family members involved in decisions about care	Regular visits facilitate access to wider support network	Confidence built in dealing with changes

Interpersonal aspects of care also featured strongly in patients' and carers' accounts: relationships between staff, patients and family carers were central to experiences of using community hospitals, and so too were relationships between patients and the wider community. Patients highlighted warm and welcoming staff, being looked after with sensitivity and respect, staff and volunteers spending time with them, being listened to, keeping their spirits up, and time taken to care for the whole person. Many respondents reflected upon how this contrasted with what they saw as a more de‐personalised patient experience associated with larger acute hospitals.

Unlike much existing literature, however, our study also highlighted the importance of distinguishing *psychological* and *social* aspects of patient experience. Social aspects of patient experience included having family, friends and wider community members close and the importance of maintaining social connections during periods of hospital treatment, rather than being distanced and isolated. The importance of social interactions *between* patients was implicit in the complaints from some that not enough was done to encourage activity and alleviate boredom.

Psychological aspects of patient experience were often wrapped up in accounts of feeling less anonymous and frightened within their community hospital than they would in an acute setting, and feeling more confident and hopeful, while also coming to terms with loss and change. Similarly, among family carers, the reassurance and reduction of stress associated with patients being cared for, often by people they knew, within a familiar, local community hospital were significant. On the other hand, this aspect also captures the shock and enormity of life events and psychological transitions, which frequently coincided with patients' use of community hospitals. While community hospitals were generally seen to build patients' confidence and physical health, a greater focus on psychological, emotional and mental health was needed.

**FIGURE 1:**
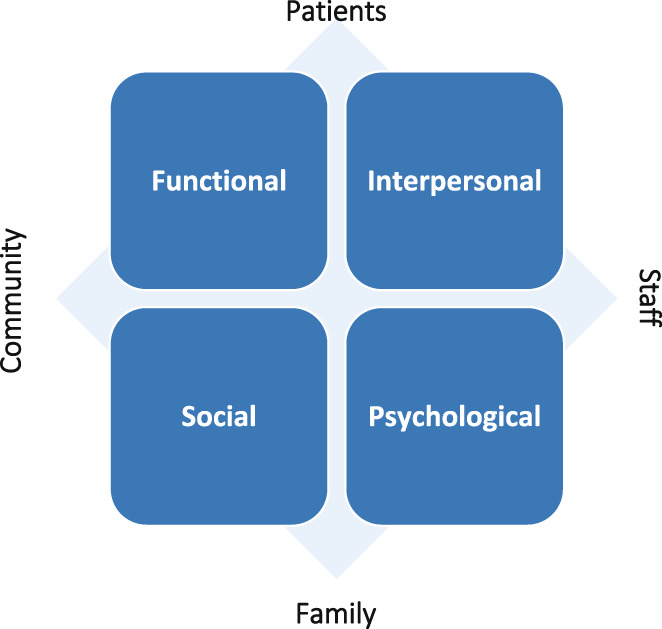
An embedded, relational model of care (first published in Davidson et al., 2019)

When considered together, the interactions between these four elements within community hospitals point to them providing an embedded, relational (rather than transactional), model of care (see Figure 1).

Personal, reciprocal, relationships between not just staff and patients, but between staff, patients, their families and the wider community were intrinsic to patients' and carers' experiences. This embedded, relational model of care was facilitated through: a closeness to home and community; the co‐location and integration of a range of intermediate, generalist, and personalised services; the small size, familiar and homely environment of community hospitals and their connection to and integration with the local community.

However, this highly valued, embedded, relational model of care cannot be assumed or taken for granted in the current policy context. A number of case study sites were facing changes as facilities became dated, services were cut back, or inpatients were drawn from an increasingly wide geographical area. Such developments were threatening some of the features of this model of care. The widening of geographical boundaries, and associated shifts towards greater provision of stepped‐down care for increasingly elderly and acute patients, had implications in some hospitals for the maintenance of the social and interpersonal aspects of care. The interpersonal aspects were also challenged by pressures on staff, exacerbated by recruitment difficulties, and were also being reshaped by the withdrawal of some GPs from community hospital medical provision (Seamark et al., 2019).

### 4.1 Limitations

There are some limitations to our study. First, while many respondents drew on comparisons with larger hospitals when recounting their experiences, our study was not designed to make direct comparisons: we suggest that this would be an important area for future study. Second, we were frustrated in our attempts to incorporate quantitative patient experience data from routine NHS sources. Finally, any assessment of service models and types requires consideration of resources and opportunity cost; while these were not formally included in our study, recent research has compared community hospital ward efficiency with the NHS acute hospital sector (Young et al., 2020).

## 5 CONCLUSIONS

Patients and family carers were overwhelming positive about their experiences of community hospitals. While they were not uncritical in their accounts of certain aspects of their care, a shared conviction that community hospitals were close to home, provided personalised and holistic care, and supported people through difficult transitions outweighed the challenges identified. Within these accounts, it is the way in which functional, interpersonal, social and psychological aspects of care interact within community hospital settings—to create an embedded, relational model of care—that contribute to these distinctive characteristics of patient and carer experience.

Our proposed, nascent, model is intended to provide a basis for future exploration and assessment of the community hospital sector and to encapsulate the range of actual and potential roles it can perform. It implies the need for Bevan's ‘imponderables’— that is, interpersonal, psychological and social criteria—to be granted greater consideration in future analysis, alongside the ongoing concern for functional aspects of care.

Further, our analysis reminds us that any assessment made about patient and carer experience is contingent on the ways that community hospitals reflect and interact with ever changing local systems and places, such that they will inevitably remain partial and contested. The patient experiences we present here are not inherent and unchanging properties of the organisations themselves, but rather features of the ways community hospitals interact with local systems, communities and individuals. Changes at any of these levels will have an impact on patient experiences.

## AUTHORS’ CONTRIBUTIONS

DD and AEP led on data collection, analysis and drafting of the manuscript. IW contributed to data analysis and drafting of the manuscript. JG was Principal Investigator and contributed to data analysis and drafting of the manuscript.

## FUNDING INFORMATION

This project is funded by the National Institute for Health Research (NIHR) Health Services and Delivery Research Programme (project number 12/177/13). The views and opinions expressed therein are those of the authors and do not necessarily reflect those of the HS&DR Programme, NIHR, National Health Services or the Department of Health.

## CONFLICT OF INTEREST

The authors have no competing interests as defined by BMC, or other interests that might be perceived to influence the results and/or discussion reported in this paper.

## ETHICS APPROVAL AND CONSENT TO PARTICIPATE

All subjects were 18 years or older. Written informed consent was obtained from all subjects. The study was granted ethical approval by the Wales Research Ethics Committee 6 (reference number: 16/WA/0021). All methods were carried out in accordance with relevant guidelines and regulations.

## CONSENT FOR PUBLICATION

All interviewees provided written informed consent for the publication of any associated data.

## Supporting information


Data S1

## Data Availability

The datasets generated and/or analysed during the current study are not publicly available due to their containing information that could compromise the privacy of research participants, but are available from the corresponding author on reasonable request.

## References

[hsc14083-bib-0001] Bevan, A. (1946). Speech on the Second reading of the NHS Bill, House of Commons, 30 April 1946. Retrived September 6, 2018 from Available online via https://www.sochealth.co.uk/national‐health‐service/the‐sma‐and‐the‐foundation‐of‐the‐national‐health‐service‐dr‐leslie‐hilliard‐1980/aneurin‐bevan‐and‐the‐foundation‐of‐the‐nhs/bevans‐speech‐on‐the‐second‐reading‐of‐the‐nhs‐bill‐30‐april‐1946/

[hsc14083-bib-0002] Bridges, J. , Flatley, M. , & Meyer, J. (2010). Older people's and relatives' experiences in acute care settings: Systematic review and synthesis of qualitative studies. International Journal of Nursing Studies, 47(1), 89–107. 19854441 10.1016/j.ijnurstu.2009.09.009

[hsc14083-bib-0003] Clarke, V. , Braun, V. , & Hayfield, N. (2015). Thematic analysis. In J. A. Smith (Ed.), Qualitative psychology: A practical guide to research methods (pp. 222–248). SAGE Publications.

[hsc14083-bib-0004] Cleary, P. , Edgman‐Levitan, S. , McMullen, W. , & Delbanco, T. (1992). The relationship between reported problems and summary evaluations of hospital care. Quality Review Bulletin., 18(2), 53–59. 1574321 10.1016/s0097-5990(16)30507-3

[hsc14083-bib-0005] Clegg, A. (2003). Older south Asian patient and carer perceptions of culturally sensitive care in a community hospital setting. Journal of Clinical Nursing, 12(2), 283–290. 12603562 10.1046/j.1365-2702.2003.00724.x

[hsc14083-bib-0006] Coulter, A. , & Cleary, P. (2001). Patients' experiences with hospital care in five countries. Health Affairs, 20(3), 244–252. 11585173 10.1377/hlthaff.20.3.244

[hsc14083-bib-0007] Davidson, D. , Paine, A. , Glasby, J. , Williams, I. , Tucker, H. , Crilly, T. , Crilly, J. , Le Mesurier, N. , Mohan, J. , Kamerāde, D. , & Seamark, D. (2019). Analysis of the profile, characteristics, patient experience and community value of community hospitals: A multimethod study. Health Services and Delivery Research, 7(1), 1–152. 30668065

[hsc14083-bib-0008] Dawson, L. (1920). Consultative council on medical and allied services, interim report: Future provision of medical and allied services. HSMO.

[hsc14083-bib-0009] Department of Health (DH) . (2006). Safety first: A report for patients, clinicians and healthcare managers. HMSO.

[hsc14083-bib-0010] Department of Health and Social Care . (2003). Keeping the NHS local: A new direction of travel. HMSO.

[hsc14083-bib-0011] Department of Health and Social Care . (2006). Our health, our care, our say: A new direction for community services. HMSO.

[hsc14083-bib-0012] Department of Health and Social Care . (2008). Delivering care closer to home: Meeting the challenge. HMSO.

[hsc14083-bib-0013] Doyle, C. , Lennox, L. , & Bell, D. (2013). A systematic review of evidence on the links between patient experience and clinical safety and effectiveness. BMJ Open, 3(1), e001570. 10.1136/bmjopen-2012-001570 PMC3549241 23293244

[hsc14083-bib-0014] Ellins, J. , Glasby, J. , Tanner, D. , McIver, S. , Davidson, D. , Littlechild, R. , Snelling, I. , Miller, R. , Hall, K. , & Spence, K. (2012). Understanding and improving transitions of older people: A user and carer centred approach. NIHR Service Delivery and Organisation programme.

[hsc14083-bib-0015] Glenn, R. , & Cornwell, J. (2011). What matters to patients? ‐ policy recommendations. NHS Institute.

[hsc14083-bib-0016] Green, J. , Forster, A. , Young, J. , Small, N. , & Spink, J. (2008). Older people's care experience in community and general hospitals: A comparative study. Nursing Older People, 20(6), 33–39. 10.7748/nop2008.07.20.6.33.c6617 18655498

[hsc14083-bib-0017] Green, J. , Young, J. , Forster, A. , Mallinder, K. , Bogle, S. , Lowson, K. , & Small, N. (2005). Effects of locality based community hospital care on independence in older people needing rehabilitation: Randomised controlled trial. BMJ, 331(7512), 317–322. 15994660 10.1136/bmj.38498.387569.8F PMC1183127

[hsc14083-bib-0018] Hadridge, P. (1997). Opportunities in intermediate care: Summary report from the Anglia and Oxford intermediate care project. DH NHS Publication.

[hsc14083-bib-0020] Jones, L. , & Exworthy, M. (2015). Framing in policy processes: A case study from hospital planning in the National Health Service in England. Social Science & Medicine, 124, 196–204. 25461877 10.1016/j.socscimed.2014.11.046

[hsc14083-bib-0021] Lappegard, Ø. , & Hjortdahl, P. (2014). Perceived quality of an alternative to acute hospitalization: An analytical study at a community hospital in Hallingdal, Norway. Social Science & Medicine, 119, 27–35. 25137645 10.1016/j.socscimed.2014.08.014

[hsc14083-bib-0022] Loudon, I. (1972). The contribution of general practitioner hospitals. The Journal of the Royal College of General Practitioners, 22, 220–226. 5074429 PMC2156653

[hsc14083-bib-0024] Payne, S. , Hawker, S. , Kerr, C. , Seamark, D. , Roberts, H. , Jarrett, N. , & Smith, H. (2007). Experiences of end‐of‐life care in community hospitals. Health & Social Care in the Community, 15(5), 494–501. 17685995 10.1111/j.1365-2524.2007.00714.x

[hsc14083-bib-0025] Pitchforth, E. , Nolte, E. , Corbett, J. , Miani, C. , Winpenny, E. , van Teijlingen, E. , Elmore, N. , King, S. , Ball, S. , Miler, J. , & Ling, T. (2017). Community hospitals and their services in the NHS: Identifying transferable learning from international developments – Scoping review, systematic review, country reports and case studies. Health Services and Delivery Research, 5(19), 1–220. 28682573

[hsc14083-bib-0026] Seamark, D. , Davidson, D. , Ellis Paine, A. , Glasby, J. , & Tucker, H. (2019). Factors affecting the changing role of GP clinicians in community hospitals: A qualitative interview study in England. The British Journal of General Practice, 69(682), e329–e335. 30803983 10.3399/bjgp19X701345 PMC6478466

[hsc14083-bib-0027] Small, N. , Green, J. , Spink, J. , Forster, A. , Lowson, K. , & Young, J. (2007). The patient experience of community hospital–the process of care as a determinant of satisfaction. Journal of Evaluation in Clinical Practice, 13(1), 95–101. 17286730 10.1111/j.1365-2753.2006.00653.x

[hsc14083-bib-0028] Small, N. , Green, J. , Spink, J. , Forster, A. , & Young, J. (2009). Post‐acute rehabilitation care for older people in community hospitals and general hospitals–philosophies of care and patients' and caregivers' reported experiences: A qualitative study. Disability and Rehabilitation, 31(22), 1862–1872. 19479496 10.1080/09638280902847002

[hsc14083-bib-0029] Trappes‐Lomax, T. , & Hawton, A. (2012). The user voice: Older people's experiences of reablement and rehabilitation. Journal of Integrated Care, 20(3), 181–195.

[hsc14083-bib-0030] Willavoys, D. , & Crowther, A. (2013). The hospitals and medical practices of Tewkesbury. Tewkesbury Hospital Leaflet.

[hsc14083-bib-0031] Williams, I. , Harlock, J. , Robert, G. , Kimberly, J. , & Mannion, R. (2021). Is the end in sight? A study of how and why services are decommissioned in the English National Health Service. Sociology of Health & Illness, 2, 441–458. 10.1111/1467-9566.13234 33636017

[hsc14083-bib-0032] Winpenny, E. , Corbett, J. , Miani, C. , King, S. , Pitchforth, E. , Ling, T. , van Teijlingen, E. , & Nolte, E. (2016). Community hospitals in selected high income countries: A scoping review of approaches and models. International Journal of Integrated Care, 16(4), 13. 10.5334/ijic.2463 PMC5354221 28316553

[hsc14083-bib-0033] Young, J. , Hulme, C. , Smith, A. , Buckell, J. , Godfrey, M. , Holditch, C. , Grantham, J. , Tucker, H. , Enderby, P. , Gladman, J. , Teale, E. , & Thiebaud, J. C. (2020). Measuring and optimising the efficiency of community hospital inpatient care for older people: The MoCHA mixed‐methods study. Health Services and Delivery Research, 8(1), 1–100. 31913588

