# Supplementary material for: ‘Localism and intimacy, and… other rather imponderable reasons of that sort’: A qualitative study of patient experience of community hospitals in England
Source: Health Soc Care Community. 2022 Nov 3;30(6):e6404–13. doi: 10.1111/hsc.14083 (PMC10092860; doi:10.1111/hsc.14083)
Supplement: Supplementary file 1 — Data S1 [file HSC-30-e6404-s001.docx]

**Community Hospitals Research study:**

**Understanding patients’ experiences**

**PATIENT DISCOVERY INTERVIEWS: TOPIC GUIDE**

**Starting the interview**

Introduce self and study.

Explain and complete consent form.

Explain that the interview is in three parts:

- **In part one:** we will ask you to tell us a bit about you and about all your experiences and stories of using this community hospital.
- **In part two:** we will explore some of the things you talked about in a bit more detail.
- **In part three:** we will ask some questions we have that we will be asking everybody who is taking part in the study, and ask you to complete a very short form with some factual information about yourself, for example your age.

**Stage 1: The patient’s story**

1. Before we begin, it would be great to know a little bit about you – your life history/ background
2. **I would like you to tell me about all your different experiences of using [X] Community Hospital, and what has been important for you.**

As you tell me your story, I won’t interrupt you – I will just make some notes. So start wherever you like and please take the time you need.

*You can use these questions to help somebody to tell their story:*

- *What happened next?*
- *How did you feel?*
- *What would have been helpful to you then?*
- *Who was with you?*
- *What was good? What could have been better?*

**Stage 2: Follow-up of patient’s story**

For stage 2 of the interview, you may follow up on any of the experience/ points/ issues raised in stage 1, that need clarifying or more information.

***You should ask questions about things in the order that the interviewee raised them.***

**Stage 3: Our questions & form (demographics)**

*Overall, the core areas we are interested in hearing about are care, treatment and support...*

If some of the factors in the drawing have not been covered in the patient’s story, you might want to ask some of the following questions, using the picture [available as a separate laminated sheet] as a prompt:


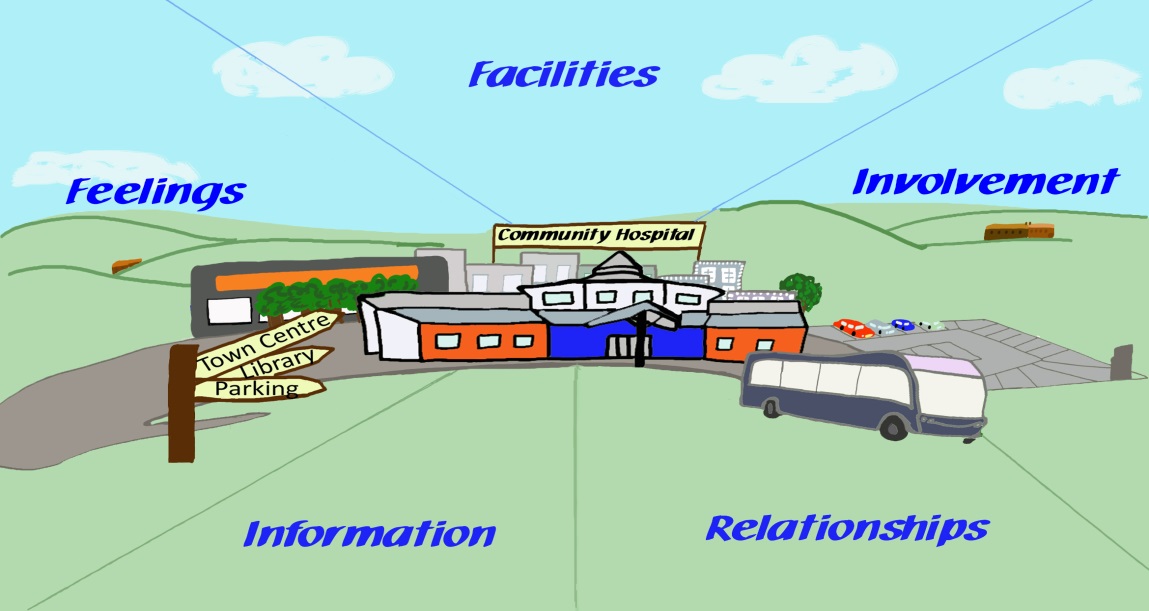


- How did you feel when you recently came to the hospital? How did these feelings affect your experience of the hospital? Do you feel the same now? (*anxiety, safe, confidence, trust).*
- How would you describe your relationships of staff at the hospital? *(family, staff, community)*
- How involved do/did you feel in decisions about your care, and how does/did this affect your experience of the hospital? *(decisions about care, in the hospital more generally)*
- What do/did you think of the facilities in the hospital, and how does/did this affect your experience? *(location, range, quality, cleanliness)*
- What kinds of information have you been given about the care, treatment and support given at [X] Community hospital? Has this affected your experience? *(about treatment, about hospital facilities, about wider services)*

**Finally two questions we are asking everyone we speak with:**

- Have you ever been involved in the hospital in any way other than as a patient, such as being a member of staff, or a volunteer?
- “What does this Community Hospital mean to you?”

**Thank you & what next**

**Complete Patient Information sheet**

**** Offer Expenses ****

**Final check:**

- - Check if participant has anything else to add
  - Thank you for taking part…
  - Check they have the information leaflet, including research team contact details
